# Intrauterine and Extrauterine Environmental PM_2.5_ Exposure Is Associated with Overweight/Obesity (O/O) in Children Aged 6 to 59 Months from Lima, Peru: A Case-Control Study

**DOI:** 10.3390/toxics10080487

**Published:** 2022-08-22

**Authors:** Valeria M. Paz-Aparicio, Vilma Tapia, Bertha Vanessa Vasquez-Apestegui, Kyle Steenland, Gustavo F. Gonzales

**Affiliations:** 1 Laboratorio de Endocrinología y Reproducción, Laboratorios de Investigación y Desarrollo (LID), Departamento de Ciencias Biológicas y Fisiológicas, Facultad de Ciencias y Filosofía, Universidad Peruana Cayetano Heredia, Lima 15102, Peru; 2 Department of Environmental Health, Rollins School of Public Health, Emory University, Atlanta, GA 30322, USA; 3 Instituto de Investigaciones de la Altura, Universidad Peruana Cayetano Heredia, Lima 15102, Peru

**Keywords:** air pollution, obesity, overweight, PM_2.5_, childhood health, intrauterine exposure, extrauterine exposure

## Abstract

There is evidence that PM_2.5_ could be obesogenic. Lima is one of the most polluted cities in South America, with an increasing prevalence of childhood obesity. This study aimed to determine the association between PM_2.5_ exposure of children aged 6 to 59 months and being overweight or obese (O/O) in a significant dataset survey. Cases were defined when weight for height Z-score (WHZ) was >2 standard deviations (SD) from the mean, for each sex. A control was defined when WHZ was between ±2 SD. We used a conditional logistic regression model to calculate the odds ratio (OR) between extrauterine and intrauterine PM_2.5_ exposure and O/O. Extrauterine PM_2.5_ exposure was evaluated as a 6-month PM_2.5_ mean prior to the survey. We found a significant association between O/O and extrauterine (OR: 1.57, 1.51–1.63) and intrauterine (OR: 1.99, 1.88–2.12) PM_2.5_ exposure for an increment of 10 μg/m^3^. The ORs increased as the quartile increased in both exposures. We observed a higher association in children aged 6–11 months (OR: 3.07, 2.84–3.31). In conclusion, higher levels of PM_2.5_ in Lima and Callao were associated with cases of O/O in children from 6 to 59 months, with the association higher for prenatal exposure.

## 1. Introduction

Environmental pollution is a public health concern affecting the population worldwide. The World Health Organization (WHO) estimated that 4.2 million premature deaths worldwide are associated with atmospheric air pollution. In addition, 91% of the global population resides in areas where pollution levels exceed WHO guidelines of ten μg/m^3^ [1].

Ambient PM_2.5_, defined as particulate material less than 2.5 microns, is one of the main atmospheric contaminants and is of anthropogenic origin in urban areas. The size of PM_2.5_ is small enough to penetrate deeply to reach the lungs, consequently impairing their functions. Moreover, high levels of PM_2.5_ in ambient air have been associated with cardiovascular, reproductive, and neurologic morbidities [2] and have also been associated with higher susceptibility to infection with SARS-CoV-2 (COVID-19) [3].

Another emerging public health problem reported globally is being overweight (Body mass index, BMI > 25, ≤30) or obese (BMI > 30) (O/O) [4]. Childhood obesity is one of the most serious public health challenges of the 21st century, and it is considered also a pandemic [5].

The evidence regarding the association between PM_2.5_ and obesity is mixed and sparse. A recent systematic review found seven studies on PM_2.5_ and obesity (three cohorts and four cross-sectional). Only two studies showed positive associations between PM_2.5_ and obesity [6]. Since that review, a prospective cohort study that analyzed polygenic risk scores for BMI found a significant positive association between particulate matter (PM_10_ and PM_2.5_) and obesity [7].

A recent cross-sectional study in children and adolescents in China concluded that for an increment of 10 μg/m^3^ of PM_2.5_, the odds ratio for obesity was 1.10 (1.03, 1.16), and the relationship was stronger in males and low economic level regions [8]. Finally, another recent study on Chinese older adults found that prolonged exposure to air pollution (PM_2.5_ and PM_10_) was positively associated with an increased risk of general obesity and abdominal obesity [9].

Air pollutants, such as PM_2.5_, can potentially act as “obesogenic” agents, as they alter the methylation of receptors and expression of genes that regulate adipogenesis [10]. Similarly, PM_2.5_ like other air pollutants acts as an endocrine disruptor, encouraging alterations in metabolism [11]. It has also been shown that prenatal exposure to PM_2.5_ is associated with an accumulation of fat in adult male mice related to an increase in food intake and an increase of neuropeptide Y (NPY), an appetite-stimulating orexigenic neuropeptide [12].

A common pathway between air pollution and overweight/obesity (O/O) is that air pollution produces an inflammatory state in the organism [13] and O/O is also associated with an inflammatory state [14]. Furthermore, this obesogenic effect may be associated with other endocrine problems. In a Chinese adult population, investigators observed a higher prevalence of cardiovascular risk factors like hypertension, type 2 diabetes, dyslipidemia, and overweight/obesity associated with long-term exposure to air pollution [15].

Lima, the capital of Peru, is a mega-city with about eleven million inhabitants and is considered one of the more polluted cities in Latin America. By 2021, Peru was ranked 26st in the world with the highest concentrations of PM_2.5_, with an average value of 29.6 μg/m^3^ [16].

According to the Demographic and Family Health Survey (DHS) in Peru, the prevalence of overweight/obesity among children aged 0–5 years old was 4.5% [17]. In Lima, Peru the co-occurrence of overweight and anemia in children is commonly found [18]. Similarly, in Lima, increased outdoor PM_2.5_ levels were significantly associated with decreased hemoglobin values and an increase in prevalence of moderate/severe anemia in children under 5 years old [19].

For these reasons, intrauterine and extrauterine exposure to increased values of PM_2.5_ may be associated with overweight/obesity in children from Lima, Peru. The present study was conducted to determine this association in children aged 6–59 months using a case-control design.

## 2. Materials and Methods

### 2.1. Study Area

Lima city is the capital of Peru on the central coast of the country near the Pacific Ocean. It comprises forty-three districts with almost ten million inhabitants, and the metropolitan area includes the neighboring constitutional province of “Callao”, with almost one million inhabitants in seven districts. For this research, we included only five districts in this province.

### 2.2. PM_2.5_ Exposure

**Figure 1:**
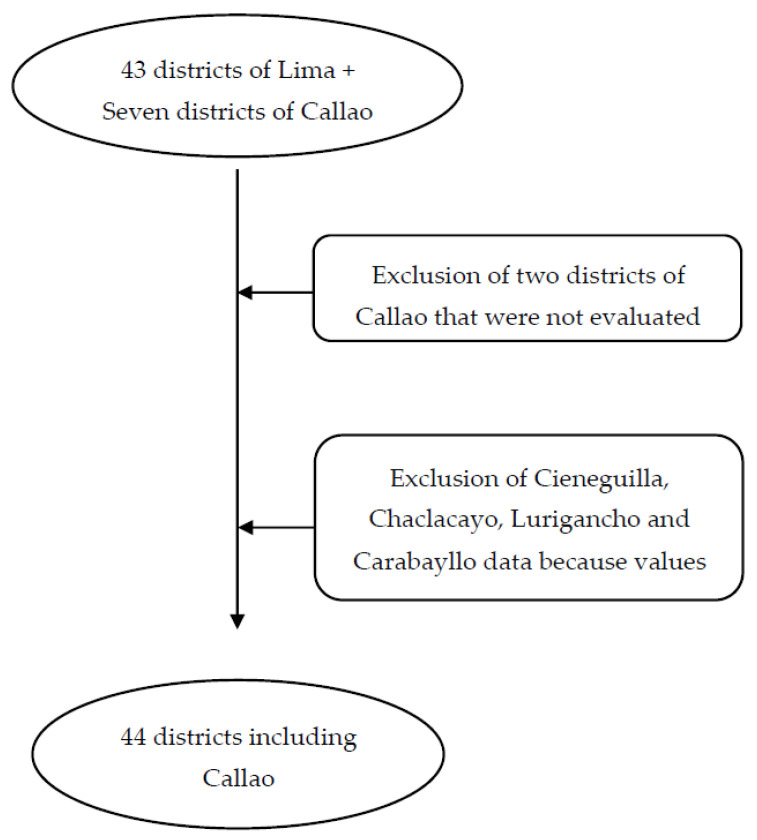
Environmental data exclusion flowchart.

PM_2.5_ was estimated daily by city district in Lima during 2010–2016 using a model that combined ground monitor data, satellite data, and a chemical-transport model [20]. The model was at a 1 km^2^ resolution. We calculated a population-weighted average across the 1-km grid for each district (*n* = 40). The model was shown to have good accuracy in relation to the ground monitors (mean difference between ground and predicted measurements = −0.09 μg/m^3^) [21]. The overall cross-validation R^2^ (and root mean square prediction error) was 0.70 (5.97 μg/m^3^) comparing predicted with observed ground-level data. The Vu et al. model [20] excluded the high-altitude districts of Cieneguilla, Chaclacayo, Lurigancho, and Carabayllo (east of Lima) because the estimates of PM_2.5_ in these districts were viewed as unreliable [20]. These districts represent just 4% of the total population. As a result, our study analyzed forty-four districts, including those from Callao (Figure 1).

### 2.3. Study Population

We conducted a case-control study using a dataset that included 441,903 children between 6 to 59 months living in Lima and Callao and attending different public health centers whose data were registered in the Nutritional Status Information System (SIEN in Spanish) by the National Center of Feeding and Nutrition (CENAN in Spanish) of the National Institute of Health (NIH) from Peru between 2012 and 2016. For this study, we studied the incidence of obesity, i.e., data from the first visit of each child under 5 years when they were found to be obese. This dataset includes information on sex, age (months), weight (kg), height (cm), district of residence, and day of the visit to the facility care center.

### 2.4. Definition of Cases and Controls

O/O in children is measured differently than in adults as children are growing and body mass index (BMI) changes by age (https://www.cdc.gov/obesity/childhood/defining.html; accessed on 22 June 2022) and gender. We defined children as O/O when weight for height Z-score (WHZ) was higher than 2 standard deviations (SD) from the mean, for each sex. A control was defined when WHZ was between minus 2 to plus 2 standard deviations [22]. O/O cases were matched on age, sex, and day of visit to the health center. WHZ is defined as below [22].

(1) $$\mathrm{WHZ}=\frac{\left(\mathrm{observed}\;\mathrm{weight}\;-\;\mathrm{WHO}\;\mathrm{reference}\;\mathrm{average}\;\mathrm{value}\;\mathrm{for}\;\mathrm{height}\;\mathrm{and}\;\mathrm{sex}\right)}{\mathrm{WHO}\;\mathrm{reference}\;\mathrm{standard}\;\mathrm{deviation}\;\mathrm{for}\;\mathrm{height}\;\mathrm{and}\;\mathrm{sex}\;\mathrm{specific}\;\mathrm{values}}$$

Cases and controls were not matched on the health center because that would in most cases be matching on residential PM_2.5_ level, which was assigned based on the Lima district of residence (*n* = 39) or Callao (*n* = 5). After restricting to the first instance of O/O, the final case-control database included a total of 32,616 O/O cases and 32,616 matched controls (1:1).

### 2.5. Variable Definitions

We obtained data for daily temperature (°C) and relative humidity (%) from the National Service of Meteorology and Hydrology (SENAMHI). An average daily temperature, relative humidity and PM_2.5_ level were calculated for each district (*n* = 44, including those from Callao). Then, for each case and control, an average for temperature, humidity, and PM_2.5_ was calculated during the 6 months before the survey visit based on their district of residence at the time of their visits. Finally, we calculated quartiles for the three environmental variables. We used data during part of 2011 to calculate exposure windows for the first health visits in 2012.

Age expressed in months was used for matching cases to controls and for the analysis. We analyzed the population as a whole and stratified analyses into age groups: 6 to 11 months, 12 to 35 months, and 36 to 59 months.

We used the poverty rate for each district in Lima and Callao, generated by the National Institute of Statistics and Informatics (INEI in Spanish) [23]. This rate considers household characteristics, household members, education, health, employment and income, pension system, ethnicity, financial inclusion, displacement, household expenses, citizen participation, and social programs per district. Data are presented as the percent of people living in poverty in a determined district. For our regression, we categorized poverty into quartiles with the first quartile being the least poor.

Finally, to calculate intrauterine exposure, we estimated the date of birth by subtracting the age (in months and days) from the date on which the child was evaluated. Subsequently, we averaged the exposure for 9 months before the birth of each child for the different districts. The Spearman correlation between intra and extra-uterine PM_2.5_ exposure was Rho = 0.90.

### 2.6. Statistical Analysis

The statistical package used for the analyses was Stata version 15 (College Station, TX, USA: Stata Press). Differences between mean values were tested with the T-student test or with the Mann–Whitney U test for normally and non-normally distributed variables, respectively.

Conditional logistic regression was used to analyze the association of PM_2.5_ with case status due to the matching of age and sex. Log ORs were estimated as a linear function of PM_2.5_ levels using as a contrast a 10 μg/m^3^ increase. Furthermore, we evaluated PM_2.5_ levels in quartiles for extra- and intrauterine exposure. The logistic model was adjusted for the quartiles of the moving average of relative humidity (%), temperature (°C) and poverty. We used the Akaike criterion (AIC) to assess model fit. Statistical significance was defined as a *p*-value < 0.05. The research was approved by the Institutional Review Board at the Universidad Peruana Cayetano Heredia.

## 3. Results

The present study included 65,232 children between 6 to 59 months, of whom 52.91% were boys. The largest population was aged 12–35 months (44.67%) at time of visit and the smallest was aged 36–59 months (21.73%). The quartiles for PM_2.5_, temperature, relative humidity, and poverty were the essentially the same in all age groups (Table 1).

Environmental PM_2.5_ 6 months prior to the diagnosis was higher for the cases than for the controls across all age groups. Temperature and relative humidity (both in the 6 months before the visit), and poverty were not different between groups (*p* > 0.05) (Table 2).

Subsequently, we evaluated intrauterine and extrauterine exposure exposition with O/O cases (*n* = 40,866; 20,433 cases and 20,433 controls) in a logistic model with controlling for temperature, humidity, and poverty (Table 3).

Table 3 reports the odds ratio for a 10 μg/m^3^ increment of PM_2.5_ as well as reporting odds ratios by quartile of exposure (separate models) with O/O for intra- and extra-uterine exposures (Table 3).

We found positive associations between both exposures and childhood overweight/obesity (*p* < 0.001). This association was stronger for intrauterine than that observed for extrauterine exposure when the ORs were compared for the highest quartile.

Increased relative humidity, temperature, and poverty were all protective against obesity.

We also analyzed the data separately by sex for extrauterine exposure (Table 4). The results did not differ by sex.

Age-specific analyses showed that the highest association of increased PM_2.5_ with O/O was observed at 6–11 months; it was moderate at 12–35 months and not apparent at 36–59 months (Table 5).

**Figure 2:**
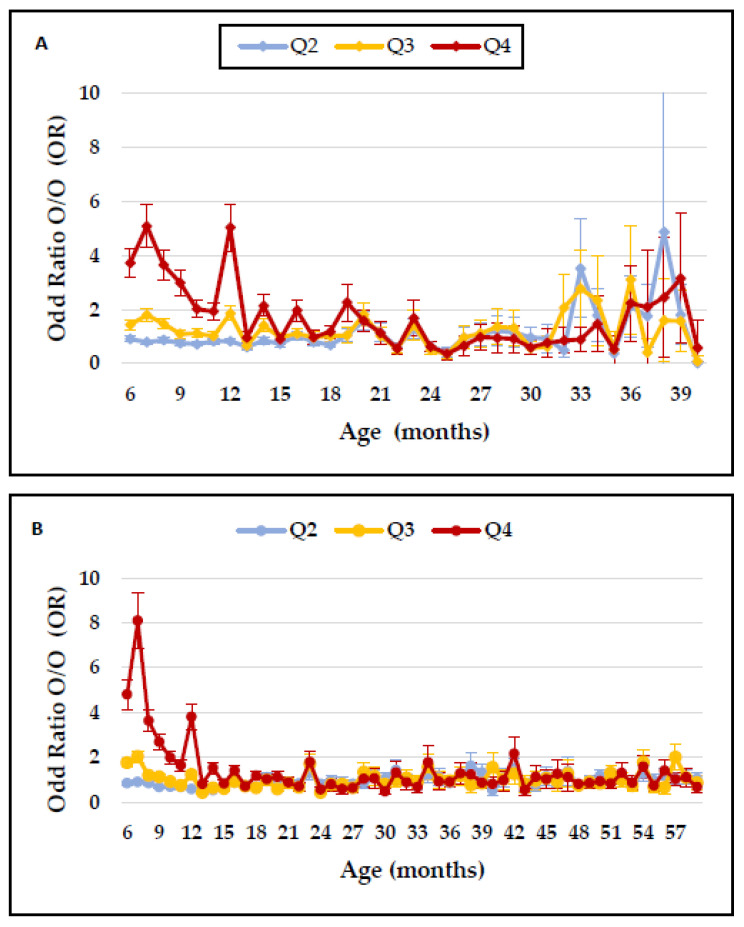
Maternal exposure in different quartiles of PM_2.5_ on O/O rate. (**A**): intrauterine exposure; (**B**): extrauterine exposure.

Figure 2A,B presents high ORs for O/O for intra- and extrauterine exposure with the highest quartile of PM_2.5_. In addition, higher ORs for O/O were observed at 31 to 39 weeks of age for intra-uterine exposure (Figure 2A) than with extra-uterine exposure (Figure 2B).

## 4. Discussion

Our study found that high exposure to PM_2.5_ was associated with O/O. These results suggest a potential increase in childhood obesity risk in children prenatally and postnatally exposed to environmental pollutants. Our results suggest that exposure to higher concentrations of PM_2.5_ in the prior 6 months including prenatally is associated with O/O cases in the child population aged between 6 to 59 months, with the strongest associations at earlier ages.

In addition, our results showed a stronger association with intrauterine exposure vs. extrauterine exposure. It has been shown previously that prenatal exposure to vehicular traffic pollution is related to accelerated weight gain in childhood [24], and it has also been associated with energy imbalance and glucose tolerance in male offspring [25]. We did not observe in our data differences between males and females.

In southern California, prenatal ambient air pollutant exposure was associated with increased weight gain and anthropometric measures from 1 to 6 months of life among Hispanic infants [26]. Our result agrees with those in that we found stronger effects at earlier ages of exposure.

The Colorado-based Healthy Start study found limited evidence of associations of prenatal exposure to ambient PM_2.5_ and O_3_ with indicators of adiposity at age 4–6 years (27). Our study results are again concordant with those, as they showed that the strongest association occurred in the youngest children, between 6 and 11 months of age, and no difference was observed in the group aged 36–59 months as reported in the study by Bloemsma et al. [27].

In another study, in Boston, USA, Fleisch et al. found no evidence for a persistent effect of prenatal exposure to traffic pollution (PM_2.5_ in the third trimester was 11.4 (1.7) μg/m^3^ on BMI trajectory from birth through mid-childhood in a population exposed to modest levels of air pollution [28]. In our study, the mean for PM_2.5_ was more than double that observed by Fleisch et al. [28], and the associations we found were driven by the higher quartile of exposure.

Regarding postnatal exposure to PM_2.5_, one study found that PM_2.5_ significantly modified the association between age and weight in males, with a positive association in children younger than 3 years and a negative association afterwards [29]. Again, the same pattern was observed in our study, with a stronger direct association at 6–11 months but no association at 36–59 months.

The evolution of adiposity with age shows that during the first year of life, fat mass increases and then decreases as age increases. Subsequently, a new ascent called “adiposity rebound” (AR) is observed [30,31]. This is a biological pattern that is displayed in different populations independent of geographic region, socioeconomic status, or sex [32]. The onset of this stage is an indicator of the future development of obesity. That is, the earlier the adiposity rebound in the child, the greater the risk of increasing fat tissue in adult life [31,33].

Normally, the average BMI rebound indicating AR was found before the age of 5 years (54–59 months) [34]. Our data suggest that high PM_2.5_ values as observed in Lima, Peru, may be a factor associated with early adiposity. Early adiposity is predictive of adult obesity [35].

We found a strong negative association of the poverty indicator on cases of O/O, where the greater the poverty, the lower the probability of overweight or obesity. The literature on this point is inconclusive. One study found that higher economic development was associated with less obese and more underweight children [36]. However, Gamboa-Gamboa et al. found a bigger prevalence of childhood obesity and overweight in children of higher socioeconomic status [37].

We also found a significant inverse association between environmental temperature and obesity when there was extrauterine exposure. These results suggest that the higher the ambient temperature, the less energy the body requires to maintain body temperature. However, Wallwork et al. demonstrated that living in warmer temperature areas increases the risk of metabolic dysfunctions like elevated fasting blood glucose, which is associated with insulin resistance [38].

It is known that obesity in the first years of life increases the probability of adult obesity, and of diseases linked to obesity such as diabetes mellitus, cancer, and cardiovascular diseases in adult life [39]. Consequently, this means a significant health risk, suggesting that Peruvian air quality environmental regulations should be revised.

The prevalence of abdominal obesity among Peruvian adults was 73.8%, being higher among women than men (85.1% and 61.1% respectively, *p* < 0.001) [40]. It is possible that exposure to air pollution prenatally and postnatally during the first years of life may contribute to this high prevalence of obesity during adulthood.

Daily physical activity has been recommended for children and adolescents to prevent overweight and obesity [41], since it has been shown that regardless of the type of exercise, physical activity maintains cellular and cardiovascular homeostasis by improving the lipid and inflammatory profiles in obese children [42]. Moreover, the probability of being obese as an adult when born to an obese father is three times higher than that born of non-obese parents [43]. Therefore, it would be best to consider parental obesity when analyzing child obesity. Not having information on child’s physical activity, parent’s obesity, and nutrition are important limitations of our study.

Another limitation of the study was that we could not evaluate other sociodemographic and health indicators that may cause childhood overweightness and obesity or that affect the association, such as type of feeding (breastfeeding, mixed feeding, solid food), diversity and composition of the diet, daily caloric intake, physical activity, sanitation, supplementation, use of medications, weight, diet and education of the mother, other diseases (metabolic, infectious and inflammatory) of the child, etc. This is because we did not have information on these variables in the dataset. However, many environmental factors affecting childhood obesity (activity levels, diet, etc.) are unlikely to be associated with air pollution and hence cannot function as confounders. Also, given the strong associations observed, we do not think they are likely to be due entirely to uncontrolled confounding.

A third limitation is that we assumed that all children were born at the end of the mother’s pregnancy. Since the exact date of birth was not recorded in the database, the calculation of intrauterine exposure was not accurate for preterm children. Furthermore, because the unit of analysis of this study is the child, another limitation is not having data on the mother’s activity during pregnancy.

We did not have data on pregnancy outcomes, which could be a limitation. PM_2.5_ is associated with lower birthweight (LBW), and therefore, we might expect that with more PM_2.5_ and more LBW babies, there would be less obesity in childhood. However, there are two reasons why these were less of a concern. First, studies linking LBW with later risk of obesity have had mixed results [44]. Second, if in fact LBW is associated with lower risk of childhood obesity, then LBW would be a mediator or intermediate variable on the pathway between PM_2.5_ and childhood obesity; one would typically not want to control for an intermediate variable in estimating the total effect of PM_2.5_ on childhood obesity.

Finally, overweight/obesity may result from catch-up growth in infants with LBW from air pollution exposure [45].

The strength of our investigation is the analysis of data obtained in a population with high environmental pollution since Lima is considered one of the most contaminated cities in Latin America [46], and we analyzed the probability of O/O attributable to PM_2.5_ exposition in a large data set as different life stages. This research is the first study that associates PM_2.5_ and overweight/obesity in Lima and Callao and shows the association of a new investigated obesogenic factor, PM_2.5_, which is a modifiable risk factor.

## 5. Conclusions

In conclusion, there are associations between elevated levels of intrauterine and extrauterine PM_2.5_ exposure with cases of O/O in children from 6 to 59 months in Lima and Callao, with the association being stronger for prenatal exposure. Furthermore, the association was stronger for children between 6 to 11 months. Potential interventions in the development of overweight and obesity at an early age is extremely important since obesity is a risk factor for developing diseases attributed to inflammation and other pathologies related to hormonal and metabolic imbalance in the future. Likewise, good control of air pollutants—with special emphasis on PM_2.5_—is important to avoid other future chronic adverse health effects in adulthood and youth ages. This information could be useful for improving public policies and strategies to control diseases.

## Figures and Tables

**Table 1:** Quartiles of poverty and environmental variables for a 6-month exposure window per age group.

O/O Aged 6 to 11	O/O Aged 12 to 35	O/O Aged 36 to 59
**PM_2.5_ (μg/m^3^) Quartiles**	**PM_2.5_ Quartiles**	**PM_2.5_ Quartiles**
Q1 (14.36–18.63)	Q1 (14.38–18.77)	Q1 (14.91–18.35)
Q2 (18.64–20.40)	Q2 (18.78–20.76)	Q2 (18.36–20.19)
Q3 (20.41–26.85)	Q3 (20.77–27.64)	Q3 (20.20–27.53)
Q4 (26.86–46.05)	Q4 (27.65–46.07)	Q4 (27.54–46.05)
**Relative Humidity (%)**		
Q1 (54.37–66.90)	Q1 (54.20–66.44)	Q1 (54.46–66.79)
Q2 (66.91–73.16)	Q2 (66.45–71.88)	Q2 (66.80–73.37)
Q3 (73.17–80.67)	Q3 (71.89–79.61)	Q3 (73.38–80.04)
Q4 (80.68–89.90)	Q4 (79.62–89.70)	Q4 (80.05–89.69)
**Temperature (°C)**		
Q1 (17.09–21.55)	Q1 (17.10–21.57)	Q1 (17.11–21.63)
Q2 (21.56–23.29)	Q2 (21.58–23.16)	Q2 (21.64–23.41)
Q3 (23.30–25.56)	Q3 (23.17–25.41)	Q3 (23.42–25.52)
Q4 (25.57–30.35)	Q4 (25.42–30.35)	Q4 (25.53–30.36)
**Poverty (%)**		
Q1 (0.17–10.48)	Q1 (0.17–10.48)	Q1 (0.17–10.04)
Q2 (10.49–16.84)	Q2 (10.49–16.84)	Q2 (10.05–15.99)
Q3 (16.85–22.80)	Q3 (16.85–22.04)	Q3 (16.00–22.04)
Q4 (22.81–29.09)	Q4 (22.05–29.09)	Q4 (22.05–29.09)

**Table 2:** Population characteristics.

Exposure	Extrauterine		Intrauterine	
Variables	Cases *n* = 32,616	Control *n* = 32,616	Cases *n* = 20,433	Control *n* = 20,433
	(Mean ± SD)	(Mean ± SD)	(Mean ± SD)	(Mean ± SD)
Weight (kg)	14.23 ± 4.36 ^a^	11.45 ± 3.23	12.29 ± 2.71	10.04 ± 2.09 ^a^
Height (cm)	82.52 ± 12.98 ^a^	81.59 ± 12.37	76.62 ± 8.86	75.89 ± 8.33 ^a^
WHZ *	2.67 ± 0.66 ^a^	0.51 ± 0.89	2.62 ± 0.61	0.54 ± 0.87 ^a^
PM_2.5_ (μg/m^3^)	23.67 ± 6.05 ^b^	22.68 ± 5.27	23.24 ± 5.40	21.80 ± 4.61 ^b^
Temperature (°C)	23.53 ± 2.49	23.43 ± 2.52	23.80 ± 2.13	23.46 ± 1.88
Relative Humidity (%)	73.10 ± 8.08	73.18 ± 8.07	72.89 ± 4.60	72.97 ± 4.65
Poverty (%)	16.10 ± 7.06	16.20 ± 6.83	16.50 ± 6.96	16.35 ± 6.67

* WHZ is the weight for height z-score value. ^a^ T-student: *p* < 0.001, cases vs. controls. ^b^ Mann-Whitney U test: *p* < 0.001, cases vs. control.

**Table 3:** Conditional regression analysis between a ten μg/m^3^ increase in intrauterine and extrauterine exposure PM_2.5_ and O/O cases in Lima and Callao.

Exposure	Overweight/Obesity *n* = 65,232 or (95% CI)	Exposure	Overweight/Obesity *n* = 40,866 or (95% CI)
**Extrauterine PM_2.5_**	**1.57 (1.51–1.63)**	**Intrauterine PM_2.5_**	**1.99 (1.88–2.12)**
**Relative Humidity (%)**		**Relative Humidity (%)**	
Q1 (54.20–66.67)	1.0	Q1 (61.18–69.54)	1.0
Q2 (66.68–72.63)	0.89 (0.83–0.95)	Q2 (69.55–73.13)	0.967 (0.89–1.05)
Q3 (72.64–80.06)	0.79 (0.71–0.89)	Q3 (73.14–75.71)	0.90 (0.81–0.99)
Q4 (80.07–89.90)	0.82 (0.70–0.96)	Q4 (75.72–86.38)	0.86 (0.77–0.97)
**Temperature (°C)**		**Temperature (°C)**	
Q1 (17.09–21.58)	1.0	Q1 (18.53–22.35)	1.0
Q2 (21.59–23.26)	0.95 (0.90–1.00)	Q2 (22.36–23.59)	0.79 (0.75–0.85)
Q3 (23.27–25.49)	0.85 (0.79–0.91)	Q3 (23.60–24.80)	0.83 (0.77–0.89)
Q4 (25.50–30.36)	0.77 (0.71–0.84)	Q4 (24.81–29.41)	0.93 (0.85–1.01)
**Poverty (%)**		**Poverty (%)**	
Q1 (0.17–10.48)	1.0	Q1 (0.17–10.48)	1.0
Q2 (10.49–16.84)	0.80 (0.77–0.84)	Q2 (10.49–16.84)	0.81 (0.76–0.86)
Q3 (16.85–22.04)	0.74 (0.70–0.77)	Q3 (16.85–22.80)	0.68 (0.64–0.72)
Q4 (22.05–29.09)	0.75 (0.71–0.79)	Q4 (22.81–29.09)	0.73 (0.69–0.78)
**PM_2.5_ Quartiles**		**PM_2.5_ Quartiles**	
Q1 (14.36–18.63)	1.0	Q1 (15.73–18.61)	1.0
Q2 (18.64–20.49)	0.83 (0.79–0.87)	Q2 (18.62–19.82)	0.86 (0.81–0.92)
Q3 (20.50–27.37)	0.97 (0.92–1.03)	Q3 (19.83–26.88)	1.24 (1.15–1.33)
Q4 (27.38–46.07)	1.52 (1.43–1.62)	Q4 (26.89–40.44)	1.99 (1.84–2.16)

CI: Confidence Interval. PM_2.5_ quartiles models are separately different from the linear PM_2.5_ exposure model reported in the first row.

**Table 4:** Conditional regression analysis for a 10 μg/m^3^ increase in extrauterine PM_2.5_ using a 6-month exposure window and O/O cases in Lima and Callao by sex.

Exposure	Male *n* = 34,514 or (95% CI)	Exposure	Female *n* = 30,718 or (95% CI)
**PM_2.5_**	**1.58 (1.50–1.67)**	**PM_2.5_**	**1.56 (1.47–1.64)**
**Relative Humidity (%)**		**Relative Humidity (%)**	
Q1 (54.37–66.67)	1.0	Q1 (54.20–66.66)	1.0
Q2 (66.68–72.64)	0.86 (0.78–0.94)	Q2 (66.67–72.60)	0.92 (0.84–1.02)
Q3 (72.65–80.07)	0.79 (0.68–0.92)	Q3 (72.61–80.06)	0.79 (0.67–0.93)
Q4 (80.08–89.70)	0.84 (0.68–1.04)	Q4 (80.07–89.90)	0.79 (0.63–0.99)
**Temperature (°C)**		**Temperature (°C)**	
Q1 (17.10–21.58)	1.0	Q1 (17.09–21.59)	1.0
Q2 (21.59–23.26)	0.94 (0.87–1.01)	Q2 (21.60–23.25)	0.96 (0.88–1.04)
Q3 (23.27–25.49)	0.83 (0.753–0.92)	Q3 (23.26–25.47)	0.87 (0.79–0.97)
Q4 (25.50–30.35)	0.76 (0.67–0.86)	Q4 (25.48–30.36)	0.79 (0.69–0.90)
**Poverty (%)**		**Poverty (%)**	
Q1 (0.17–10.48)	1.0	Q1 (0.17–10.48)	1.0
Q2 (10.49–16.84)	0.83 (0.78–0.88)	Q2 (10.49–16.84)	0.78 (0.72–0.83)
Q3 (16.85–22.04)	0.76 (0.71–0.82)	Q3 (16.85–22.04)	0.71 (0.66–0.76)
Q4 (22.05–29.09)	0.77 (0.72–0.83)	Q4 (22.05–29.09)	0.72 (0.67–0.78)

CI indicates, confidence interval at 95%.

**Table 5:** Odds ratios for O/O for a 10 μg/m^3^ increase in PM_2.5_ for extrauterine exposure using a 6-month exposure window by age group.

Exposure	O/O Aged 6 to 11	O/O Aged 12 to 35	O/O Aged 36 to 59
	*n* = 21,918 or (95% CI)	*n* = 29,146 or (95% CI)	*n* = 14,174 or (95% CI)
**Extrauterine PM_2.5_**	3.07 (2.84–3.31)	1.31 (1.24–1.38)	0.98 (0.91–1.01)
**Relative Humidity (%)**			
Q1	1.0	1.0	1.0
Q2	0.87 (0.77–0.99)	0.81 (0.73–0.89)	1.07 (0.91–1.25)
Q3	0.72 (0.59–0.89)	0.71 (0.60–0.84)	1.00 (0.79–1.27)
Q4	0.75 (0.56–1.00)	0.79 (0.63–1.00)	0.89 (0.65–1.23)
**Temperature (°C)**			
Q1	1.0	1.0	1.0
Q2	0.97 (0.88–1.07)	0.94 (0.87–1.02)	0.94 (0.84–1.07)
Q3	0.83 (0.73–0.94)	0.81 (0.73–0.90)	0.94 (0.81–1.09)
Q4	0.71 (0.60–0.83)	0.72 (0.63–0.82)	0.94 (0.78–1.14)
**Poverty (%)**			
Q1	1.0	1.0	1.0
Q2	0.72 (0.66–0.78)	0.84 (0.79–0.91)	0.88 (0.80–0.98)
Q3	0.58 (0.53–0.64)	0.76 (0.70–0.81)	0.96 (0.86–1.07)
Q4	0.70 (0.64–0.77)	0.74 (0.69–0.80)	0.95 (0.85 -1.06)
**PM_2.5_ Quartiles**			
Q1	1.0	1.0	1.0
Q2	0.79 (0.72–0.86)	0.78 (0.73–0.84)	1.03 (0.92–1.14)
Q3	1.27 (1.16–1.40)	0.78 (0.72–0.85)	0.98 (0.87–1.11)
Q4	3.33 (2.98–3.72)	1.11 (1.01–1.21)	0.99 (0.87–1.12)

Each age group is a model adjusted by temperature, relative humidity, and poverty. PM_2.5_ quartiles models are different from the linear PM_2.5_ exposure models. Age group expressed in months.

## Data Availability

Data can be available at request.
